# *Rurbanity*: a concept for the interdisciplinary study of rural–urban transformation

**DOI:** 10.1007/s11625-023-01331-2

**Published:** 2023-05-20

**Authors:** Ellen M. Hoffmann, Nikolaus Schareika, Christoph Dittrich, Eva Schlecht, Daniela Sauer, Andreas Buerkert

**Affiliations:** 1 Organic Plant Production and Agroecosystems Research in the Tropics and Subtropics, Universität Kassel, Steinstrasse 19, 37213 Witzenhausen, Germany; 2 Social and Cultural Anthropology, Georg-August-Universität Göttingen, Theaterstrasse 14, 37073 Göttingen, Germany; 3 Human Geography, Georg-August Universität Göttingen, Goldschmidtstrasse 5, 37077 Göttingen, Germany; 4 Animal Husbandry in the Tropics and Subtropics, Universität Kassel and Georg-August-Universität Göttingen, Steinstrasse 19, 37213 Witzenhausen, Germany; 5 Physical Geography, Georg-August-Universität Göttingen, Goldschmidtstrasse 5, 37077 Göttingen, Germany

**Keywords:** Assemblage, Interdisciplinarity, Rural–urban transformation, Social–ecological system, Sustainability, Urbanisation

## Abstract

Along with climate change, population growth, and overexploitation of natural resources, urbanisation is among the major global challenges of our time. It is a nexus where many of the world’s grand challenges intersect, and thus key to sustainable development. The widespread understanding of urbanisation as a successive and unidirectional transformation of landscapes and societies from a rural to an urban state is increasingly questioned. Examples from around the globe show that ‘the rural’ and ‘the urban’ are not only highly interdependent, but actually coexist and often merge in the same space or livelihood strategy. Our concept of *rurbanity* provides an integrated theoretical framework which overcomes the rural–urban divide and can be operationalised for empirical research. *Rurbanity* is the next stringent step following the gradual widening of previous concepts from urban-centred approaches through the emphasis on urban peripheries to attempts of abolishing any distinction of a rural environment and acknowledging the highly dynamic nature of globalising urbanisation. Building on complex systems theory and assemblage thinking, our concept explores complementary aspects of the distinct epistemic worldviews dominating the natural and social sciences. Within this theoretical frame, we derive four analytical dimensions as entry points for empirical research: *Endowments and Place*, *Flows and Connectivity*, *Institutions and Behaviour*, and *Lifestyles and Livelihoods*. Two examples illustrate how these dimensions apply, interact, and together lead to a comprehensive, insightful understanding of *rurban* phenomena. Such understanding can be an effective starting point for assessing potential contributions of *rurbanity* to long-term global sustainability.

## Introduction

Urbanisation is accelerating globally. Today, 56% of the world’s population lives in urban areas, and by 2050 the proportion of urban population is expected to reach 67% (World Bank 2020). Between 2018 and 2050, the total urban population will grow by an estimated 2.5–3 billion people. This world-scale urban transformation has been addressed by various UN reports (UN Habitat 1996; UN 2019) and has led to the declaration of an Urban Age, or a Planetary Urbanisation, which exemplifies the Anthropocene in its geographical form (Swyngedouw 2014). Currently, nearly 90% of the urban growth is taking place in Africa and Asia (UN 2018). Cities, particularly those in the Global South, will be increasingly important agents for humanity to thrive on Earth, but at the same time among the areas most affected by losses in ecosystem services, depending on how global urbanisation is shaped.

Urbanisation, with its effects on the environment, is certainly one of the major global challenges of our time (WBGU 2016), along with climate change (IPCC 2021), population growth (UN 2017), and overexploitation of natural resources that approach planetary boundaries (Rockstrom et al. 2009; Steffen et al. 2015). It is a nexus where all of these grand challenges intersect and often a driver of future trends in development (While and Whitehead 2013). This makes rural–urban transformations a key arena for achieving the Sustainable Development Goals on which the global community has agreed (UN 2015; Elmqvist et al. 2021). Particularly in low-income and lower middle-income countries, cities are facing political, social, economic, and ecological challenges and are struggling to meet the needs of their growing populations, including housing, transportation, energy systems, and other infrastructure, as well as employment and basic services such as education and health care (Zhang 2016).

As their populations grow, cities also grow spatially, by combinations of building densification, high-rise, and lateral sprawl (Angel et al. 2021; Marconcini et al. 2021). Surface sealing for urban infrastructure occurs mostly, and with increasing speed, at the expense of prime agricultural land (Bren d’Amour et al. 2016). On the other hand, according to FAO statistics on land use, urban areas still comprise less than 3% of the total land area, whereas agriculture, including croplands and pasture, accounts for 36% (47 million km^2^ of 130 million km^2^; FAO 2022). Similar shares were estimated for the year 2000 in an earlier study based on remote sensing data (Ramankutty et al. 2008) and in the analysis of the spatial history of human land use by Ellis et al. (2021). World Bank statistics (2022) classified 86% of the total land area (112 million km^2^) as rural in 2010, although the classification of a given area as rural or urban certainly has serious ambiguities. To urbanists, ‘the rural’ often constitutes just the stage on which urbanisation unfolds, especially as the research and policy community addressing rural development is largely divorced from the urban arena and operates within other scientific disciplines and policy domains (van Vliet et al. 2020).

The physical dimension of urbanisation and the social–ecological and political challenges that arise from it have reignited the scientific discussion about the relationship between the rural and the urban. Concepts based on the rural–urban dichotomy are increasingly questioned as to whether they are still suitable to adequately explain the highly intertwined rural–urban settlements, social–ecological systems, and societal arrangements that emerge in the outskirts of cities and in metropolitan regions (McGee 1991; Marshall et al. 2009; Brenner and Schmid 2014; Brenner and Katsikis 2020). Likewise, the presumption that transition proceeds one-directionally towards the urban, and that it ceases or stops once a completely urbanised stage is reached, is increasingly refuted (Gutierrez-Velez et al. 2022). Since the turn of the twentieth century, when scholars in Europe began to formally study the spatial planning of cities, research focused strongly on metropolises and megacities, whereas rural areas were neglected. Even more, the in-between remained an entirely blind spot in the discourse. Koolhaas (2014) pointed to the urban bias by stating that “our current obsession with only the city is highly irresponsible, because you cannot understand the city without understanding the countryside”, or even stronger: there is “no [rural] outside left to conquer” (Tzanninis et al. 2021, p. 229) and “rural and urban livelihoods and lifestyles can blend together to the point where ‘the rural’ and ‘the urban’ become indiscernible.” (Guiterrez-Velez et al. 2022, p. 3). To describe the new configurations and relationships between the urban and the rural under globalising conditions, including its spatial structures and social, political, and cultural articulations, we elaborate a concept termed ‘*rurbanity*’, with its underlying process of ‘*rurbanisation*’. We argue that it is far better suited than concepts of rural–urban gradients, peri-urban fringes, or rural–urban interfaces to capture the being and becoming of entangled rural and urban spatial structures, material flows, institutions, forms of social practice, and lifestyles.

The development of this concept has to engage the natural and social sciences in a way that is suitable for analysing the heterogeneous structure and the dynamic transformation of *rurban* space and the *rurban* phenomena contained therein. We suggest two theoretical frameworks as particularly helpful to this end: that of social–ecological systems and that of assemblages. The first is well established in interdisciplinary research; the latter is used in the social sciences, and here particularly in research focusing on the link between society and technology and environment (Lowenhaupt Tsing 2015). Using examples from previous and ongoing research, we propose our concept as an innovative analytical framework to integrate different domains of scientific knowledge and outline a general roadmap for its empirical applicability. We conclude with a brief outlook on implications of *rurbanity* for sustainability.

## Analysis of existing conceptual framings for rural–urban relationships

**Fig. 1:**
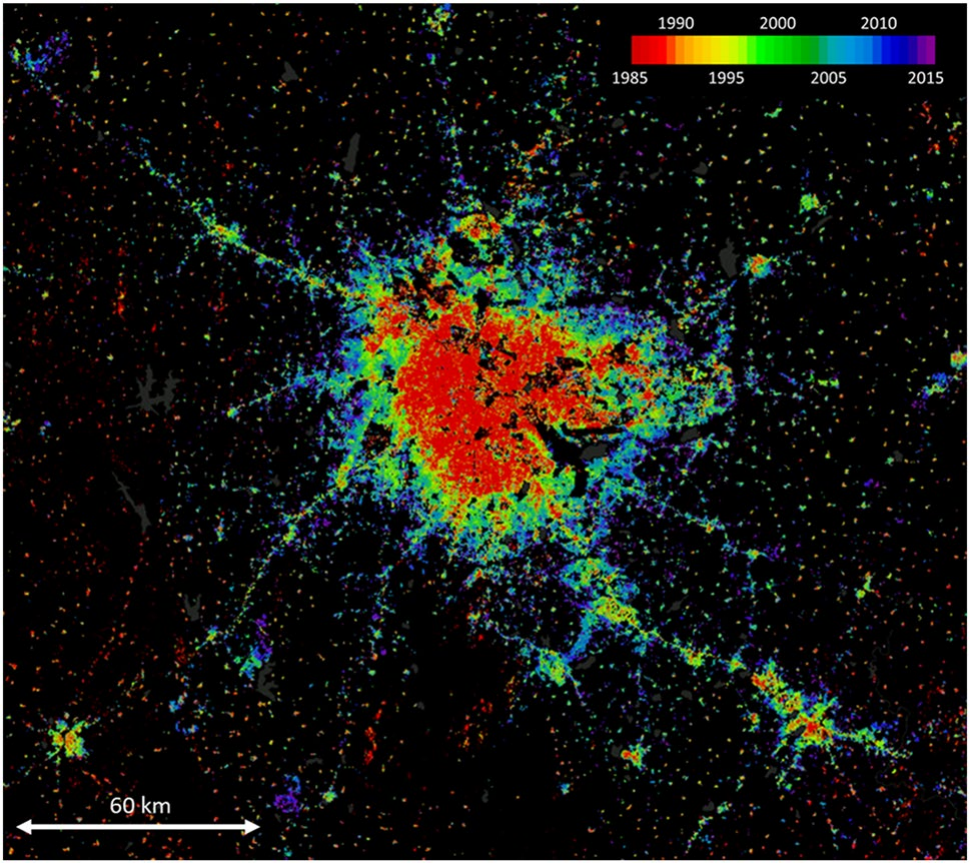
Expansion of built-up area around the southern Indian megacity of Bengaluru from 1985 to 2015 (WSF-Evolution, DLR). Multiple and diverse examples of *rurbanity* emerge in the diffuse rural–urban fringes

Global urbanisation cannot be spatially delineated and has an impact far beyond urban centres and agglomeration areas. As the example of the megacity of Bengaluru in southern India shows (Fig. 1), cities are increasingly diffusing at their edges and the rural–urban interface is characterised by a mosaic-like structure of spatial units of different functions, agricultural and non-agrarian activities (Hoffmann et al. 2021). It is criss-crossed by a closely knit network of infrastructures and flows of people, resources and goods, energy, information, knowledge, and innovation. New geographies of ‘citylands’ are emerging, and everyday routines, values, and rationalities are subject to profound changes (Roy 2009). This new dialectic between the urban and the rural requires a redefinition of the relationship between both realms that goes well beyond dualistic urban–rural notions. In the following, we briefly review a number of concepts that seek to capture socio-spatial reconfigurations beyond the traditional urban–rural dichotomy.

### Gradients and indices

Nearly 200 years ago, von Thünen (1826) proposed a model of concentric rings of different agricultural land use around an urban centre determined by economic functions. His concept lay the foundation for many attempts to describe and define the impact of a city on its rural surroundings by mathematical models. Most recently, Chang et al. (2022) presented a linear mechanistic model based on gravity forces related to functional requirements for satisfying urban demands. Earth observation and land use science (van Vliet et al. 2020) have also been applied to develop quantitative indices for the degree of urbanity in relation to the distance from a central city (Schlesinger and Drescher 2013; Hoffmann et al. 2017). This approach is useful as a descriptive tool for temporary phenomena, but has little explanatory power for drivers of transformation.

### Peripheries and interfaces

In the early 1990s, McGee pointed to spaces in the extended environments of major Asian cities, where non-agricultural and agricultural forms of land use and settlement coexist and are highly linked to each other. He referred to these spaces as ‘desakota’, a term he derived from the Indonesian words ‘desa’ (village) and ‘kota’ (city; McGee 1991). He distinguished three types of desakota, based on differential combinations of land use patterns, economic development, and population dynamics. Indovina (1990) referred to the urbanised landscape of the city and its immediate hinterland as ‘città diffusa’ and Sieverts (1997/2001) as ‘Zwischenstadt’. The widely used concept of ‘peri-urban’ refers to the urban fringe and the geographic edge of cities: “as a place, it refers to the movement of goods and services between physical spaces and to the transition from rural to urban contexts, as a process, it refers to an interface between rural and urban activities, and finally, as a concept, to institutions and perspectives” (Marshall et al. 2009, p. 3). Many of those ideas are also subsumed under the term ‘suburbanisation’ (Tzaninis et al. 2021). There are different approaches of conceptualising the peri-urban. Place-based approaches, understanding the peri-urban as a heterogeneous conglomeration of rural–urban features lying between cities and countries (Cadėne 2005), have to be distinguished from flow-based conceptualisations which emphasise the “flows of produce, finance, labour, and services” and the influence of “processes of rapid economic, sociological, institutional, and environmental change” (Halkatti et al. 2003, p. 149). The dynamism of change inherent in peri-urban spaces is evident in the use of the term ‘zone’ or ‘rural–urban interface’. Finally, Allen et al. (2006) provide a working definition of the peri-urban as instances where rural and urban features coexist, in environmental, socio-economic, and institutional terms.

### Continuous rural–urban landscapes

The worldwide rural-to-urban transition encompasses a vast spectrum of settlement conditions, from sprawling megacities with their peri-urban satellite towns, to regional centres and small towns, no matter whether classified as urban or not (Brenner and Schmid 2014). “Increasingly, the urbanisation process has become a global condition rather than simply a ‘way of life’ that is confined to certain types of settlement space as compared to others” (Brenner and Schmid 2014, p. 747). Taking these ideas further, the authors call for a decentring perspective that redirects attention from the cities to what was formerly perceived as ‘rural hinterlands’, as those are nowadays an integral part of continuous, rural–urban ‘operational landscapes’ (Schmid 2018; Brenner and Katsikis 2020). The urbanisation patterns observed in different case studies have proven to be highly variegated, complex, and context dependent (Schmid et al. 2017). In a comparative analysis, Schmid et al. (2017) derived a typology of different urbanisation processes described as ‘popular urbanisation, plotting urbanism, multilayered patchwork urbanisation, or laminar urbanisation’, among others. Their proposed terminology aimed at taking account of the spatial characteristics as well as the socio-political drivers that shaped the case studies.

From the perspective of urban political ecology, Tzanninis et al. (2021) identified four major challenges in gaining a comprehensive understanding of rural–urban transitions: the methodological city-ism, a neglect of Southern contexts, the rift between academia and the policy arena, and the inclusion of non-human elements (primarily nature and ecology) in urbanisation concepts. Overcoming these constraints would reveal rural–urban landscapes worldwide as a ‘more-than-urban continuum’ to which there is no longer any outside. They also emphasise that “nowadays some of the most dynamic socio-political changes happen in the periphery”, and “it is in the sprawl where sustainability, community, and the urban have to be found” (Tzanninis et al. 2021, p. 243). So far lacking in their approach, however, is an operational and empirical approach to their insightful claims.

### Temporary urbanism and alternative-substitute place-making

With a strong focus on the Global South, on the interplay between formal and informal governance, and on the dynamics of transformation processes, Andres et al. (2021) addressed some of the above-mentioned challenges: “African cities and Global South cities should be conceptualised as the outcome of layers of planned interventions combined with alternative-substitute place-making that represents different forms of ‘permanent impermanence’” (Andres et al. 2021, p. 30). The authors described urban planning as a macro-scale intervention, and place making as a micro-scale practice of neighbourhood residents that interact and mingle in the peri-urban space. In contrast to urban planning paradigms, the informal practices of place making stand out due to flexibility combined with short-term and everyday adaptability. In theoretical terms, the authors pointed out that “a system of systems approach is required to account holistically for the different connected components underpinning social, economic, and environmental well-being” (Andres et al. 2021, p. 30). Their conclusions, however, were limited to practical recommendations for improving urban planning.

### Critique

While by no means complete, all of the alternative concepts highlighted above acknowledge that the established categories of the urban and the rural are insufficient to describe contemporary lived realities which feature multiple elements of diversity, uncertainty, and self-organisation. The examples were also selected to show how concepts have progressively widened over time, from urban-centred approaches, through emphasis on urban peripheries, towards attempts to abolish any distinction of a rural environment and to acknowledge the highly dynamic nature of urbanisation. All of these approaches, however, argue from a specific disciplinary perspective, such as economics, land use science and agricultural science, political ecology, or urban planning. Though most of them aim at integrating different dimensions of urbanisation, they still lack a coherent framework from which entry points for empirical research could be derived. One reason for this may be the differing, but often tacit epistemological worldviews that guide research traditions in natural compared with social sciences.

## The concept of “*rurbanity*”

Fifty years ago, the French sociologist Henri Lefebrve already described urbanisation as a ‘total’ phenomenon that has suspended the rural–urban divide and thus the historical categories of urban and rural (Lefebvre 1972). While we accept that the rural and the urban continue to (co)exist, we argue that they are organised in specific entanglements that we call ‘*rurban*’. We understand *rurban* as the continuous reconfiguration of material flows, practices, contexts of meaning, and spatial structures. Accordingly, our analysis of ‘*rurbanisation*’ requires, first, the simultaneous and equal attention to rural and urban practices, spatial structures, and imaginaries. Secondly, the *rurban* substantiates the ambiguity of the categories rural and urban and critically re-evaluates their associated attributions. The analysis of ‘*rurbanity*’ therefore implies a permanent (re-)positioning within complex and highly dynamic relationships between the rural and the urban. By defining this state as an independent object of study, constituted in the dialectical gap between the categories of rural and urban but in itself an object, our concept makes an ontological contribution to that field which can be operationalised for interdisciplinary research.

The fusion of the words ‘urban’ and ‘rural’ has been previously reported. The terms *rurban* and *rurbanisation* can be tracked back to the sociologist Sorokin, who referred to them in 1929 as a terminological invention of C. P. Galpin in 1918 (Dymitrow 2017). Lacour and Puissant (2007) took up the desakota concept when analysing the changing relationship between the rural and the urban under conditions of globalisation. They described a process of ‘ruralisation of the urban’ and the result of this development as a state of *‘rurbanity’*.

The term *rurban* was revived and used more coherently when several Indian scholars adopted it to describe urbanisation processes in India, albeit with slight differences in their specific definitions (Revi et al. 2006; Gupta 2015; Kolhe and Dhote 2016). Revi et al. (2006, p. 58) defined ‘*rurbanism*’ as “a process integrating the urban with the rural, so that there is a co-evolution of the countryside and the city that is embedded within it.” In 2016, the term even lent its name to a nation-wide rural development programme in India, ‘India’s National *Rurban* Mission’ (https://rurban.gov.in; Singh and Rahman 2018). By means of concerted policy directives, this programme envisions developing clusters of settlements that preserve and nurture the essence of rural community life with a focus on equity and inclusiveness, without compromising with facilities perceived to be essentially urban in nature, thus creating *‘rurban* villages’. While this prominently illustrates how a novel term can promote a new vision that finally manifests itself in a real-world policy, it is also an example of a purely top-down state agency. Accordingly, this strand of literature talks about *rurbanism*, whereas in our concept we prefer the noun *rurbanity*. The suffix-ism, derived from the Latin-ismus, denotes a doctrine, a principle or a faith system. The suffix-ity, on the other hand, forms abstract nouns from adjectives, referring to a quality, a state, measure, or distribution of something, thus meaning a ‘condition or quality of being’ (http://www.uefap.com).

**Fig. 2:**
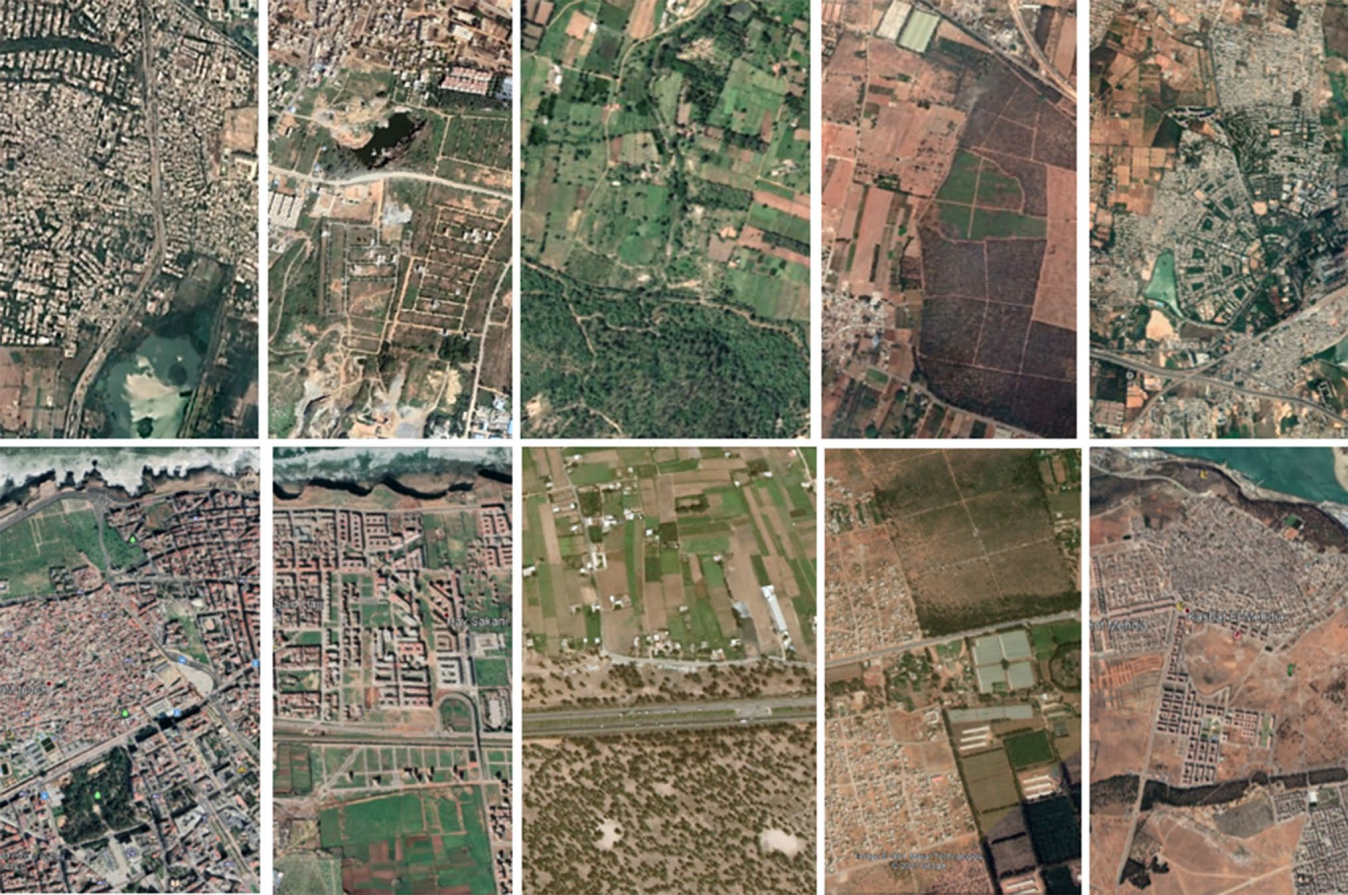
Google Earth satellite images of the Bengaluru Metropolitan Area in southern India (above) and the Rabat–Kenitra Corridor in Morocco (below) demonstrating striking similarities in land use patterns across distant locations and cultural settings

**Fig. 3:**
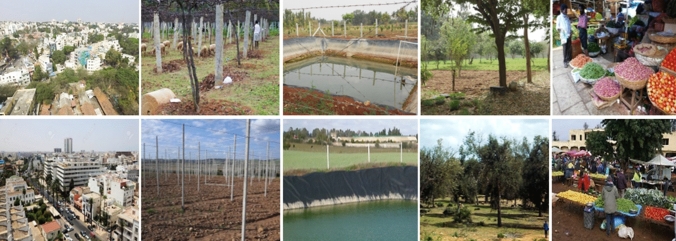
Ground photographs of r*urban* locations in the Bengaluru Metropolitan Area in southern India (above) and the Rabat–Kenitra Corridor in Morocco (below) demonstrating striking similarities in sceneries across distant locations and cultural settings

Our focus, thus, is the environmentally, socially and culturally productive co-presence of urban and rural elements and practices—in the widest sense of the term, including matter, relations, and ideas—within a shared space that is being structured by that very co-presence. Our elaborated concept of *rurbanity* points to the fact that, when rural and urban elements come together, intermingle, and assemble, they give rise to characteristic spatial, social and environmental phenomena that share a number of similarities, irrespective of the local context. This is illustrated in Figs. 2 and 3 by the comparison of satellite images (Fig. 2) as well as photographs on the ground (Fig. 3), showing different locations in the Greater Bengaluru region (India) and the Rabat–Kenitra region (Morocco). The mix of residential and commercial building structures, parks, roads, street-lining trees, and agricultural fields in both locations demonstrates that similar *rurban* patterns are emerging in geographically, socially, and politically unrelated regions. Our concept makes it possible to lay out a roadmap how the emergence and development of these phenomena can be analysed when taken up as an object of interdisciplinary research.

## Linking the concept of *rurbanity* with social–ecological system analysis and assemblage thinking

By using the concept of *rurbanity*, we emphasise the connection between heterogeneous elements in a shared space. Moreover, the connection between these elements and the patterns within which they are arranged appear stable in the sense that stability is achieved through the constant persistent creation of the emergent (Deleuze and Guattari 1987; Protevi 2006). In physics and chemistry, such scenarios are theoretically described as ‘dissipative structures’ (Prigogine 1978) that persist far from thermodynamic equilibrium by constantly absorbing energy ‘from the outside world’ to maintain their order. This is a basic principle that actually applies to all living systems and enables them to grow in size and complexity, to adapt, and to evolve (Holling 2001; Kurakin 2010). Therefore, we consider the concept of *rurbanity* as a contribution towards, not a critique of, general system theory (von Bertalanffy 1950). More specifically, to usefully conceptualise *rurbanity,* we draw on two theoretical approaches that share a number of commonalities, but also have important differences: social–ecological systems (a specification of complex adaptive systems) and assemblage thinking (Spies and Alff 2020).

The merit of both is that they invite researchers to seek connections between items situated within different ontologies. For example, there is a straightforward language to describe how agricultural soil reacts to the addition of water, but there is no such language to describe how water reacts to an institution or a cultural value and vice versa. However, by looking at a space of formation and transformation that is sourced from two broad directions—the urban and the rural—with an enormous number of heterogeneous elements of completely different ontological status, we conceptualise *rurbanity* as a constellation and process of being and becoming. *Rurbanity* associates elements that so far have not been studied as being part of one encompassing system, but still become part of a process of arranging and rearranging of items that, at first sight, would not go together. *Rurbanity* thus points to a phenomenon that defies modernist ideas of planning from above as well as scientific routines of knowing and predicting through established system modelling and extrapolation. In this regard, it fits the assemblage concept that emphasises heterogeneity (multiplicity), practice (events), emergence, creativity, and openness over boundedness, unity, and equilibrium.

The term ‘assemblage’, derived from the French term ‘agencement’ (Deleuze and Guattari 1987; Buchanan 2015; Law 2004), is different from hybridisation. Hybridisation refers to the process of mixing two things to produce new forms, for example ‘creole’ forms of culture (Pieterse 1993). An assemblage is “some form of provisional socio-spatial formation […] composed of heterogeneous elements that may be human and non-human, organic and inorganic, technical, and natural” (Anderson and McFarlane 2011, p. 24). The elements of an assemblage thereby maintain their individual identities. The assemblage is constantly creating, recreating, and transforming the arrangements of the relations connecting its empirically perceivable items (De Landa 2006). It is important to add that in the complex reality of social assemblages, they are permeated with multiple asymmetries concerning power and access to all kinds of resources.

*Rurbanity*, viewed through the framework of social–ecological systems, highlights the connectedness of the material with the social, of the social with the symbolic, and of the social–material with the spatial, forming habitats that are marked by a high degree of direct and planned human, socially, and culturally constituted intervention. At the same time, *rurbanity* is also usefully studied as an assemblage at work to explore why some connections are enabled while others become dysfunctional. This question, though, cannot be answered once and for all because, following the notion of assemblage and knowledge built from prior empirical research, we have to understand *rurbanity* as an arrangement that is the spatio-temporally specific condition for its own re-arrangement. If we understand *rurbanity* as a state of being and becoming, its study requires a conceptual language that is capable of grasping phenomena that are not fixed, are transient, have no clear-cut boundary between an inside and an outside, are more than one thing at a time, and do not only follow linear causalities.

One of the processes that characterises *rurbanity* and would be highlighted by an assemblage approach is ‘bricolage’, as described by Claude Lévi-Strauss (1966). An engineer would carefully procure the raw materials and the most appropriate tools needed for a specific purpose or project, and thus depend on the availability of those. A ‘bricoleur’, by contrast, would take whatever is available and use it in the best possible manner. His “set of tools and materials (…) is always finite and is also heterogeneous (…). They each represent a set of actual and possible relations; they are 'operators' but they can be used for any operations of the same type” (Lévi-Strauss 1966, p. 18). This idea of bricolage implies a high motivation for the local recycling of resources, whereas engineering might rather rely on external inputs. The application of such principles in urban development has been termed ‘urban tinkering’ (Elmqvist et al 2018), or ‘jugaad’ in India (Radjou et al. 2012). The description of several examples showed that the actual implications, however, remain to be assessed in each specific context.

Our concept of *rurbanity* goes beyond bricolage*,* because we understand that depending on the circumstances also other forms of ordering heterogeneous items are at work. The concept highlights the contingency in the formation and transformation of rural–urban spaces that do not follow a preconceived plan, but emerge in a generalised way from multiple creative quests to gain utility by combining things one has not asked for but found. It also highlights the fact that that in this process normative orders of what can be combined with or used for what are easily and elegantly transcended. Thus, a balcony can become a barn, a sewage conduit an irrigation system, and a kinsman a salaried employee.

The theory of complex adaptive systems is applicable in the natural sciences (Prigogine and Stengers 1984; Dooley 1996; Holling 2001), as well as in social sciences such as economics and governance research, emphasising that social and environmental dimensions are intertwined and inseparable (Liu et al. 2007; Ostrom 2009; Ostrom and Cox 2010; Preiser et al. 2018). With its holistic aspiration, this kind of systems thinking is well suited to analyse *rurbanity*, particularly as the concept strives to make systems-oriented ecological analysis a fundamental component of the study of the profound societal and historical processes upon which the creation of urban space is based. Since both approaches, complex adaptive systems and assemblage, share the interest in human–environmental research, combining them bears a high potential for meaningful syntheses, and utilisation in integrative, interdisciplinary research (Spies and Alff 2020). The entanglement of society and nature, relations and dynamics as constitutional factors, path dependency, emergence, and self-organisation are important guiding principles in both schools of thinking. Our concept of *rurbanity* applies this approach to the research field of rural–urban transformations and exemplifies an attempt to realise the synthesis potential pointed out by Spies and Alff (2020).

## Operational entry points for analysing *rurbanity*

To translate this highly abstract theoretical conception of *rurbanity* into empirically based interdisciplinary knowledge production, an operational framework is needed. This operational framework has to integrate the material, the social, and the cultural dimensions of *rurbanity*; it also has to allow for both a system-oriented and a process-oriented analysis of the phenomenon. We suggest building this operational framework by defining four analytical dimensions or perspectives as entry points (Boone et al. 2014; Schmid et al. 2017) that, in combination, elucidate the structure (the being) and the transformational dynamics (the becoming) of *rurban* phenomena: (1) *Endowments and Place*, (2) *Flows and Connectivity*, (3) *Livelihoods and Lifestyles,* and (4) *Institutions and Behaviour.*

The perspective of *Endowments and Place* picks out the place-based properties of a *rurban* phenomenon, narrowing down the scope from the global scale of urban footprints to specific regions of highly dynamic rural–urban transformation, and determines their characteristic material and immaterial resources and assets at a given moment in time. It allows us to ask which features these regions share to make them focal points of *rurbanity*. Endowments may comprise natural resources such as raw materials, water and fertile soils, or ecosystem services, but also human-made infrastructures such as housing, transport and communication networks, or human and social capacities such as a well-educated labour force or cultural achievements of a population.

*Flows and Connectivity* addresses the regional to global networks through which these material and immaterial resources flow in multiple forms of exchange that operate at different spatial and temporal scales (Karg et al. 2019, 2023). Such flows, which rely on the connectivity within and between multiple networks at different scales, offer choices to *rurban* actors and thus confer high flexibility towards them, either to adapt and enhance resilience or to find innovative solutions and effect transformation. Spotswood et al. (2021) show that a number of non-human species make use of such opportunities, too. However, such networks of resource flows may also increase the vulnerability of certain groups from a local to the global level, for example, through the use and accumulation of contaminated resources in food production.

*Livelihoods* describe the means of securing necessities for life, such as occupation, access to resources and information, reliance on social networks, and supporting institutions (Stienstra and Lee 2019). The perspective of *Livelihood* is particularly suitable to capture the dynamics of *rurbanity* and to meaningfully integrate our interdisciplinary research on these dynamics. While originally devised for the study of rural economies that goes beyond the local and accounts for their wider, indeed global connections (Ellis 2000; Jones and Craswell 2004), the concept highlights exactly those properties of socio-ecological processes that we deem crucial to studying the dynamic and multi-dimensional processes of the making of *rurbanity*. *Lifestyles* depict the way of life that defines and reinforces self-identity. Following Adler ([1933] 2008), we understand lifestyle as a creative force with which people try to overcome their shortcomings, express themselves, and value what they need. *Lifestyles* are expressed by and associated with occupation, socio-economic status, consumption levels. The perspective of *Lifestyles* also highlights the role of cultural systems for particular forms of sociality, identity, and practices of social distinction (Walters 2006). Taking particular interest in processes of transformation, we are also aware that lifestyles may exhibit enormous inertia that affects socio-ecological dynamics in *rurbanity*.

The perspective of *Institutions and Behaviour* is drawn from two sources: (a) the economic school of New Institutionalism (North 1990) which builds on earlier institutional thinking in economics (Veblen 1919); and (b) the anthropology of politics as practice (Bailey 1969). Institutions are socially devised instruments of regulation and governance that exist in various forms including laws, norms, rules of conduct, or moral values. They are established and maintained by a collective that can be the state, but also a local community as Ostrom (1990) argued against Hardin (1968); they can be formal as well as informal. While institutions award roles and identity to individual and collective agents (March and Olsen 1989), the perceptions, preferences, and risk attitudes of individual actors, their expectations, decision-making, and daily routines are described by *Behaviour* (Simon 1959; Gächter and Herrmann 2009). This perspective thus helps to qualify the notions of individual choice, on the one hand, and utility maximisation, on the other, and thereby assess their impact on the sustainability of resource use. While New Institutional Economics interprets institutions to be a result of rational actors' collaborative efforts to minimise transaction costs (North 1990), social anthropology has emphasised two features of *Institutions and Behaviour* that particularly fit the *rurban* situation. First, actors are not simply constrained by institutions (Hardin 1968); they can sometimes manipulate them in highly creative ways according to their interests in competitive situations of negotiation and conflict (Bailey 1969; Swartz 1966). Second, old institutions are often not replaced by new ones, but pile up in a historical process so that conflicting actors have a heterogeneous, complex and contradictory set of rules at hand that they strategically exploit for their individual advantage (Benda-Beckmann 1981; Benda-Beckmann 1997).

In combination, the four perspectives *Endowments and Place*, *Flows and Connectivity*, *Institutions and Behaviour*, and *Livelihoods and Lifestyles* thus capture the biophysical as well as socio-cultural dimensions of *rurbanity* across multiple scales. Since they are interrelated in many ways, their joint application bears a high potential to carve out synergies in the comprehensive analysis of *rurban* phenomena. To show how they apply to generate knowledge through empirical research, we turn to two examples.

## The concept of *rurbanity* in operation

*Rurbanity* is related to spatial features, but not to a specific location. It can be used to analyse transformations in highly contested peri-urban fringes as a response to economic and administrative conflicts, as well as to balcony or rooftop gardening in densely populated urban centres as an expression of cultural values. In West Africa, it may also refer to the telecoupled unsustainable intensification of agriculture in remote desert oases as a response to market demands in coastal cities, which allows business and consumers to externalise negative consequences of urbanisation on ecosystem services (Liu et al. 2013; Fastner et al. 2023 unpublished). We present two examples of *rurban* phenomena from our empirical research in India and West Africa, which were analysed previously in a conventional, disciplinary context. We demonstrate where other frameworks fall short in explaining these phenomena, and how the concept of *rurbanity* can help to explain the unity of seemingly incompatible systems of practice, knowledge, and meaning.

### Dairy cows in urban India

**Fig. 4:**
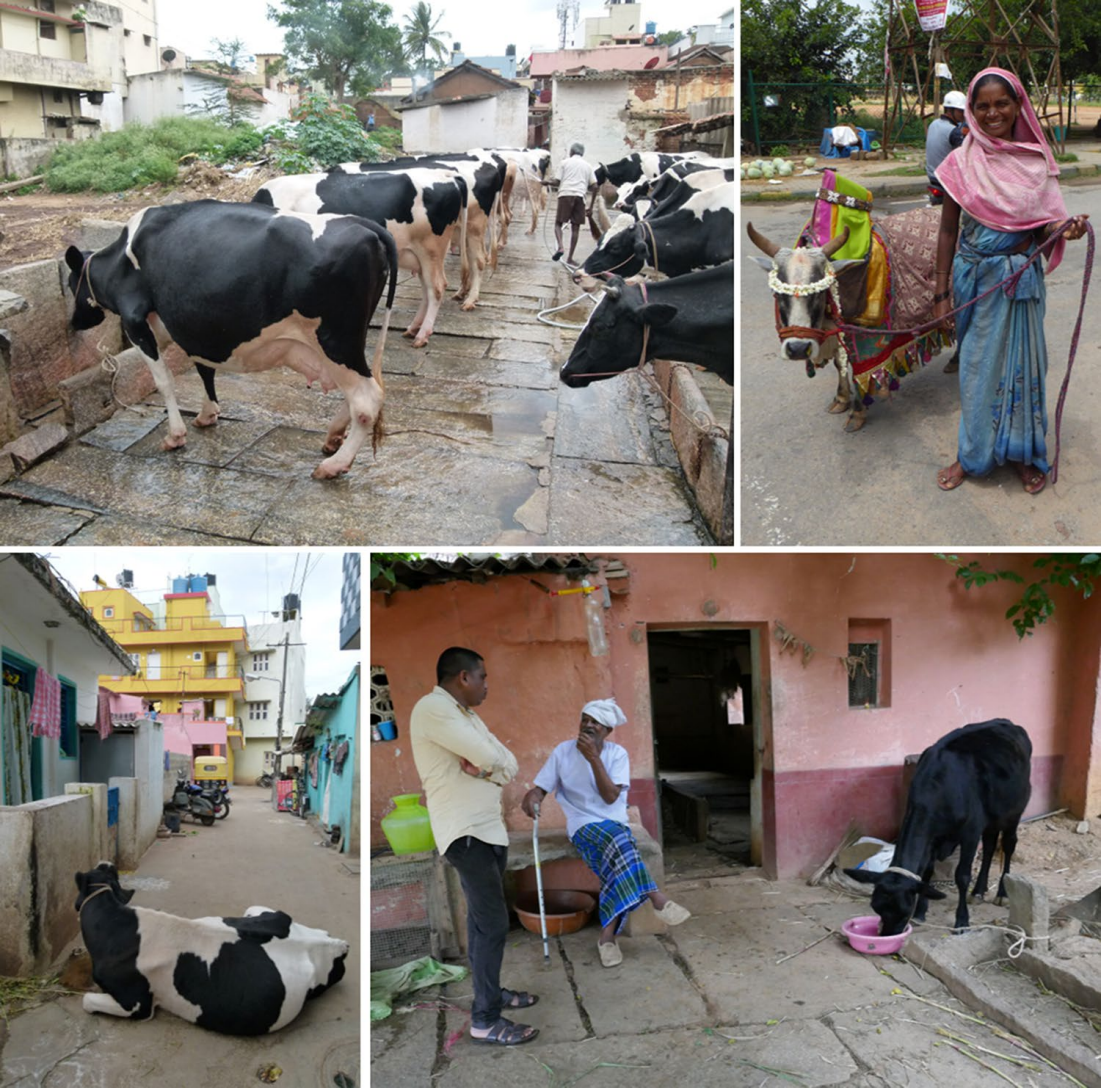
*Rurbanity* exemplified by the close relationship between cows and humans in Bengaluru, India: dairy production in an inner-city animal shed (above left), a cow presented as cultural icon (above right), and dairy cows kept by households in different urban neighbourhoods (below)

For thousands of years, keeping cattle close to humans was part of India’s socio-cultural traditions. Some decades ago, as part of India’s ‘Milk Revolution’, Holstein Frisian and Jersey cattle breeds were introduced into the subcontinent and interbred with local breeds to enhance milk yields in small locally interconnected producer units (Kurien 2007). Recent research in Bengaluru, a megacity with more than 12 million inhabitants and capital of the south Indian State of Karnataka, has shown that an estimated 5000 buffaloes, 6000 indigenous cattle, and 75,000 crossbred cattle are kept in the agglomeration (Prasad et al. 2019). The majority of these cattle are kept by individual households in small-scale herds of up to five animals (Fig. 4). Their milk yield is either sold directly to inner-city consumers or to the dairy cooperative Karnataka Milk Federation (Reichenbach et al. 2021a).

Understanding this system as a *rurban* phenomenon allows us to analyse its features against the background of originally rural skills, traditions, and belief systems of the animal holders in an environment that has been quickly overgrown by the urban structure of a burgeoning megacity (Pinto et al. 2020a). Recent data show that in the inner city, milk yield and body weight of cattle is higher due to better management and access to high-quality feedstuff, such as vegetable waste from neighbouring households (Reichenbach et al. 2021b). This entails lower values of enteric methane emission per litre of milk in the peri-urban zone (Pinto et al. 2020a) than in rural areas. Furthermore, inner-urban cattle suffer less from heat stress (Pinto et al. 2020b) and enjoy good hygiene management as derived from low infestation with gastrointestinal parasites (Pinto et al. 2021). At the same time, the very clean but rigid granite and cement flooring of inner-city housing environments leads to an increased frequency of mechanical injuries (hook lesions and lameness) of cattle kept in the densely inhabited areas (Pinto et al. 2020b).

The concept of *rurbanity* provides a better understanding of how agricultural traditions of animal keepers from formerly rural communities are closely intertwined with their new urban-based capital-oriented *Livelihood* in which they sell and purchase cows as required by cash needs, market opportunities, and abrupt changes of the social–ecological environment, such as the COVID-19 pandemic (Alam et al. 2022). It also highlights that Bengaluru’s animal sheds, as a formerly rural unit now adapted to the necessities of the city, are washed with tap water several times per day to prevent odour from disturbing urban neighbours. Animal excreta are thus flushed away rather than recycled as manure to cropland as typical on Indian rural farms (Reichenbach et al. 2021a). From the perspective of *Endowments and Place*, it is evident that housing space and pasture area for the animals are extremely contested in the inner city, and pasture area is continuously declining at the city fringes (Pinto et al. 2020b; Reichenbach et al. 2021a). However, from the perspective of *Institutions and Behavior*, negative environmental impacts such as (ground) water pollution through manure-derived nutrients and enteric methane emissions of ruminants are regulated by Indian laws at the local to national level (Arora et al. 2017), appropriate housing, handling, and feeding of cattle is governed by ethical norms deeply rooted in Hindu culture, which are currently also discussed as guidelines for cow-care at the global level (Phillips 2021). Alternatively, aspects of farm animal welfare and ethics could also be targeted from the multifunctionality perspective of agricultural production, or rather, ‘coordination and organisation’ at the farm, cooperative, and societal level, and in this relate to the ‘institutional jointness’ advocated by Hagedorn (2007).

When addressing the *Flows and Connectivity* dimension, it becomes evident that the cattle-keeping families take advantage of the specific opportunities offered by the urbanising environment, for example by happily accepting organic food waste as cattle feed from neighbours, who, in turn, buy the animals’ milk (Reichenbach et al. 2021b). In addition to the direct flow of materials, this practice is also an example of the social connection between milk-producing farmers and food waste-dispensing neighbours who may be pursuing a wasteful lifestyle (Ganguly 2017). From the perspective of *Institutions and Behaviour* we have seen that grazing cattle on roadsides and open construction sites, and collecting fodder from lakeshores is a widely used strategy (Reichenbach et al. 2021a; Alam et al. 2022) building on common property principles that traditionally support *Livelihoods* in rural India (Gaur et al. 2018). Beyond their contribution to the cattle-keepers’ income, the animals provide food products, and employment along the pre- and post-harvest value chain (Younas 2013).

At the same time, cattle keeping in Bangalore supports the *Lifestyles* of non-agricultural middle-class families who, besides buying and consuming milk, source online shops such as www.amazon.in to order well-packed cow manure as fertiliser for urban roof top gardening (Wikström 2017) or for the Hindu pooja ritual. The collection and composting of animal manure and other organic materials that fuel this flow of materials indirectly connect dairy farmers with, for example, *rurban* rooftop gardeners, and also provide livelihoods for poor people in the important but precarious informal waste-recycling sector. Connecting this sector’s expertise in waste collection and separation with the currently emerging formal ‘urban mining’ sector to enhance overall material recycling and reuse could reduce negative impacts on remote areas where raw materials are typically being sourced, strengthen flows and connectivity within the *rurban* arena, and potentially contribute to the emergence of new cooperative behaviour (Arora et al. 2017).

### Cattle fattening in scrap-recycling yards in urban Ghana

**Fig. 5:**
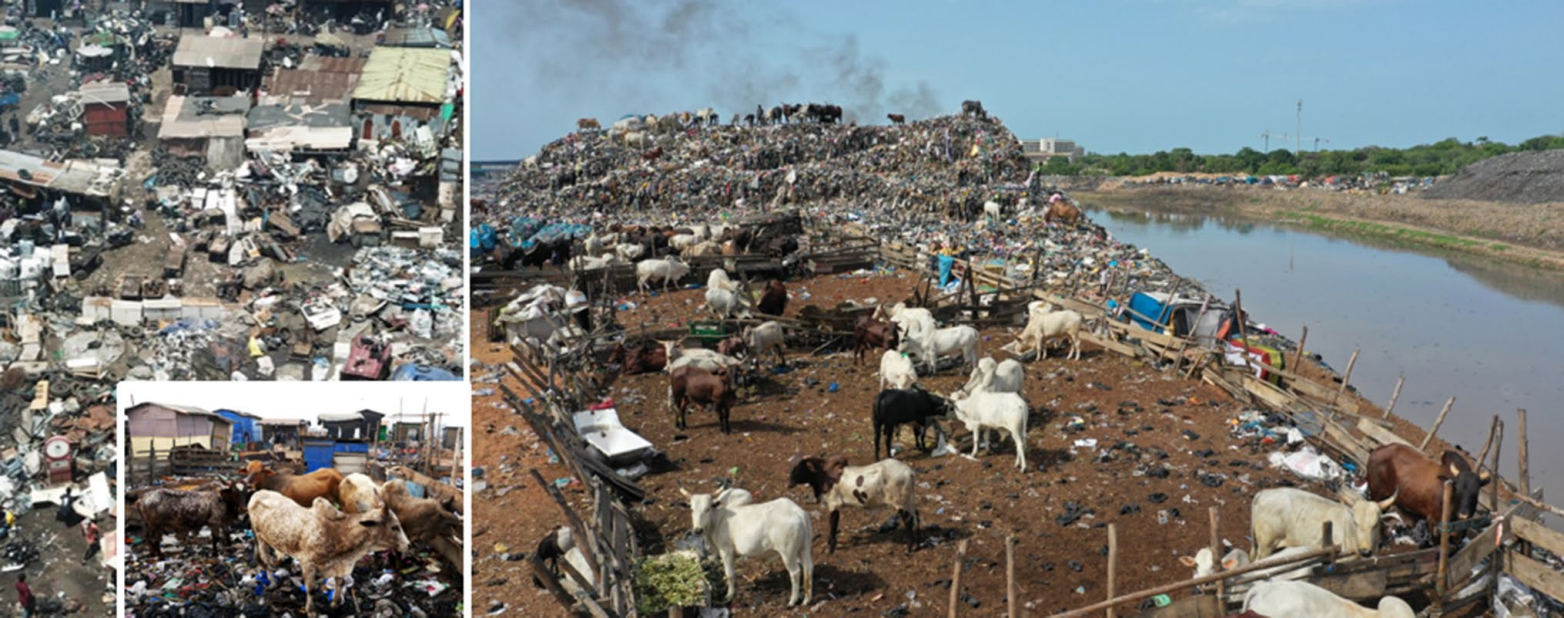
The e-waste-recycling site of Agbobloshie in Accra, Ghana, just before its dismantling in July 2021 (left) and the neighbouring slum of Sodom to where *rurban* cattle fattening activities have shifted in April 2022 (right)

During the last decade, Agbobloshie, an e-waste-recycling area in Ghana’s capital Accra, became widely known as one of the world’s most contaminated areas where thousands of new settlers from rural areas dismantle, under the most ecologically and socially difficult conditions, broken electrical appliances and other scrap materials such as old tyres in search of metals to be sold (Oteng-Ababio 2012; Adanu et al. 2020). It is much less known that in the same location, hundreds of freely grazing cattle and sheep are kept for milking or are stabled in corrals to be fattened for meat production (Fig. 5). This example can be understood as a *rurban* assemblage with apparently unrelated elements entering into various relations by sharing the same (physical and social) space.

By applying the perspective of *Endowments and Place* along with that of *Institutions and Behaviour*, it becomes clear that use rights of space at Agbobloshie are heavily contested. They are allocated by a traditional chief who maintains strict order and resolves conflicts with little respect for formal municipal or federal laws. Agbobloshie and the related slum of Sodom have their own institutions, health system, and security system, governed by what fits best to the needs of the scrapyard and animal fattening business with its local, regional, and global material flows. The same applies to the workforce, which is in high demand for the many activities on the dumpsite. Here, too, it is the chief and his assistants who give permission on where to dismantle tyres or computers and where to raise cattle. The *Livelihood* of cattle buying, fattening, and selling is completely in the hands of newly arrived rural migrants coming largely from the Kumasi area of south Ghana who continue their formerly pastoral livestock economy by investing the capital gained from waste recycling in cattle keeping under new social–ecological conditions. The perspective of *Flows and Connectivity* can shed light on interactions between the flow of electronics from within Ghana to a central repair and dismantling area, the long-distance import of e-waste from the Global North, the inflow of people who establish flows of cattle feed from urban vegetable markets, and of concentrates from the city fringes and rural hinterlands. These ecologically critical livestock production activities take place in the immediate neighbourhood of well-paying middle-class consumers, for whom regular consumption of high-quality meat in burger restaurants or at traditional grill stands is one expression of their urban *Lifestyle* (Latino et al. 2020). The multifaceted livelihood arrangement at Agbobloshie is governed by strict rules and hierarchies that defy spatial constraints and concerns about the risks of water and soil contamination for consumers. It is so resilient against disruptions in environmental or legal conditions that the entire animal husbandry waste-recycling complex shifted to the nearby slum of Sodom within days after the municipal government had unexpectedly decided to bulldoze Agbobloshie in early August 2021 in the wake of speculators’ interests in developing these grounds together with those of an adjacent onion market into a luxury residential area (Akese et al. 2022). Should these new land development visons ever gain ground, they would showcase the severe consequences on local well-beings by shortcutting contaminant flows in a highly contested and subsequently contaminated *rurban* environment.

While the examples and reflections above focus on human actors, it is undisputed that non-human beings such as plants and animals also inhabit the *rurban* space. These organisms can constitute positive endowments, for example through providing ecosystem services such as clean air, shade, or pollination, as well as threats, for example through potential disease transmission or attacks on humans (Perry et al. 2020; Coman et al. 2022; Divakara et al. 2022). Furthermore, the complexity and aspirations of modern *rurban* life and lifestyles of human inhabitants also affect habitats and survival of remote non-human organisms through telecoupled processes such as deforestation, expansion of agricultural land, sand and mineral mining, and unidirectional material flows to *rurban* areas that may operate over hundreds of kilometres (Friis and Nilsen 2014; Karg et al. 2016, 2019). The latter, in return, render *rurban* spaces into valuable mining areas for all sorts of ‘waste’ from demolished buildings, electronic devices, and end-of-life vehicles to discarded plastics and organic materials (Arora et al. 2017).

## Conclusions and outlook

As a concept to better understand the interdependence and dynamics of environment, society, structures, and processes that shape rural–urban transformations, *rurbanity* enables us to overcome the increasingly blurry divide between the urban and the rural in a rapidly urbanising world. Flows of material, people, cash, and knowledge similarly shape very different places, creating strikingly congruent patterns of *rurban* land use and production systems. Against the background of social–ecological systems theory and assemblage thinking, we have elaborated the concept to a coherent analytical framework and developed four perspectives that can be adopted for qualitative as well as quantitative research and thus inspire combining different epistemologies. They provide familiar entry points for researchers from different scientific disciplines, but also leverage potentials for synergies along the course of work. Similar to the object of study, the research approach itself can thereby become dynamic and adaptive, assemble diverse elements in a bricolage, and amalgamate them to new methods for a more holistic understanding of complexity. Tied to resources, but also open to dynamically adjust and readjust to multifaceted conditions, *rurbanity* is exposed to, and coproduces local, regional, and global grand challenges. At the same time, however, it is a breeding ground for potential solutions that foster local, regional, and global sustainability.

This concept implies that social–ecological entities in a *rurban* mosaic constantly create multiple transient solutions for securing their existence. Some of those may be guided by myopic profitability, others by deeply engraved cultural values or visions for the future. Independent of their time horizon, they may be more or less sustainable. Though in a perpetual state of nascency and thus highly dynamic, they aggregate to constitute a permanence of the impermanent at higher scales, which may grow into a pervasive state of *rurbanity*. Exploring the mechanisms of self-organisation that would channel arbitrary, indeterminate development paths towards long-term global sustainability certainly warrants further research at the interface of interdisciplinary theory building and empirical research.

## Data Availability

Due to the conceptual character of our paper, our work uses a theoretical approach developed by the author team and relies on the cited literature. Where we presented examples, we refer to data collected in other project contexts. These projects are ongoing, and the data collected there are protected by a Memorandum of Agreement among the project members, such that they are not (yet) publicly available. Parts of the experimental data are available from the authors upon reasonable request and upon permission of all concerned project members.
